# Prognostic Value of Thrombocytopenia in Myelodysplastic Syndromes After Hematopoietic Stem Cell Transplantation

**DOI:** 10.3389/fonc.2022.940320

**Published:** 2022-07-11

**Authors:** Hong Wang, Jiaqian Qi, Xueqian Li, Tiantian Chu, Huiying Qiu, Chengcheng Fu, Xiaowen Tang, Changgeng Ruan, Depei Wu, Yue Han

**Affiliations:** ^1^ National Clinical Research Center for Hematologic Diseases, Jiangsu Institute of Hematology, The First Affiliated Hospital of Soochow University, Suzhou, China; ^2^ Institute of Blood and Marrow Transplantation, Collaborative Innovation Center of Hematology, Soochow University, Suzhou, China; ^3^ Key Laboratory of Thrombosis and Hemostasis of Ministry of Health, Suzhou, China; ^4^ State Key Laboratory of Radiation Medicine and Protection, Soochow University, Suzhou, China

**Keywords:** hematopoietic stem cell transplantation, myelodysplastic syndrome, prognosis, thrombocytopenia, risk factor

## Abstract

Prolonged isolated thrombocytopenia (PT) is a common complication affecting the outcome of stem cell transplantation. In this study, we undertook a real-world study of 303 myelodysplastic syndrome (MDS) patients who received allogeneic hematopoietic stem cell transplantation (HSCT) between December 2007 and June 2018. 28.4% of MDS patients suffered from PT after HSCT. Survival analysis indicated that PT was associated with worse overall survival (OS) in MDS patients. The 2-year and 5-year OS in MDS patients with PT after HSCT were 49% and 47%, significantly worse than that of 68% and 60% in patients without PT (P=0.005). For RFS, patients with PT did not have an increased risk of disease relapse (P=0.964). After multivariate adjustment, PT was proved to be the independent risk factor associated with the worse OS (HR 1.49, 95% CI 1.00-2.21, P =0.048). We further analyzed risk factors associated with the occurrence of PT in MDS patients. Multiple logistic regression identified grade II-IV aGVHD, extensive chronic GVHD, hemorrhagic cystitis, and CMV activation as significant risk factors for developing PT. Among these variables, the Odds Ratio (OR) of grade II-IV aGVHD was the highest (P =0.001, OR: 2.65, 95% CI: 1.51-4.64). These data indicated the prognostic value of PT in MDS after HSCT. The identification of risk factors for PT may help improve patient management and lead to the design of effective treatment strategies.

## Introduction

Allogeneic hematopoietic stem cell transplantation (Allo-HSCT) is the only curative treatment for patients with myelodysplastic syndrome (MDS). Prolonged isolated thrombocytopenia (PT) is a frequent complication after transplantation, includes primary poor platelet graft function (PPGF) and secondary failure of platelet recovery (SFPR) (1, 2). PT has been suggested to be associated with an increased platelet transfusion requirement and poor overall survival following allo-HSCT (2–6).

The mechanisms underlying development of PT after HSCT are complex, and are usually categorized into impaired platelet production and increased platelet destruction (7). Several potential risk factors for PT after HSCT have been suggested, including graft-versus-host disease (GVHD), doses of infused CD34+ cells, disease status, cytomegalovirus (CMV) infection, and donor-specific antibodies (6, 8–11). Reports from Kong Y and her colleagues demonstrated that impaired bone marrow vascular microenvironment and aberrant T cell responses in immune microenvironment may contribute to the occurrence of PT after HSCT (12–14). They also proposed that disease type, especially diagnosed as MDS, was an independent risk factor for SFPR (12).

We undertook a single-center real-world study in the Chinese population, focusing on characteristics of PT in MDS patients. The purpose of present study was to evaluate the prognostic impact and defined the potential risk factors for PT in MDS patients after HSCT.

## Materials and Methods

### Patients and Study Design

303 consecutive MDS patients who received allo-HSCT in the First Affiliated Hospital of Soochow University between December 2007 and June 2018 were included in our study. Patients’ age, gender, WHO classification, IPSS and IPSS-R risk, donor type, conditioning regimen, stem cell source, disease status, HLA typing, ABO blood group, GVHD prophylaxis, and transplant related complications were recorded. Informed consent was obtained from all patients or from their immediate family before data was collected. All protocols conformed to the guidelines of the ethics committee of Soochow University and the Declaration of Helsinki. All patients were followed until September 2019 or death.

### Conditioning Regimens in Allo-HSCT

Myeloablative conditioning (MAC) regimens were applied in most cases, while all other patients received reduced intensive conditioning (RIC) regimens. For HLA matched sibling donor transplant (MSDT), MAC regimens comprised administration of semustine (250mg/m^2^, day −10), cytarabine (2g/m^2^/d, days −9 to −8), busulfan (3.2 mg/kg/d, days −7 to −5), and cyclophosphamide (1.8g/m^2^/d, days −4 to −3). For HLA matched unrelated donors transplant (MUDT) and haploidentical donors transplant (HIDT), patients received a MAC regimen identical to the MSDT regimen except for receiving a higher dose of cytarabine (4 g/m^2^/d, days −9 to −8). Patients receiving MUDT also received hydroxycarbamide (80mg/kg, day −10). The RIC comprised fludarabine (30 mg/m^2^/d, days −10 to −6), cytarabine (1.5 g/m^2^/d, days −10 to −9), busulfan (3.2 mg/kg/d, days −8 to −6), cyclophosphamide (1.0 g/m2/day, days −5 to −4), and semustine (250 mg/m2/day, day -3). Additionally, Rabbit ATG (Genzyme Polyclonals S.A.S, Lyon, France), ATG-F (Fresenius Biotech GmbH, Munich, Germany), or porcrine ALG (Wuhan Institute of Biological Products Co., Ltd., Wuhan, Hubei, China) was given to patients receiving MUDT and HIDT for GVHD prophylaxis. The regimens were: ATG 2.5mg/kg/day, for four days; ATG-F 5mg/kg/day, for four days; ALG 15mg/kg/day, for four days. For a small number of patients who received MSDT, a lower dose of ATG (2.5mg/kg/day, for two days) or ATG-F (5mg/kg/day, for two days) was used.

### Definitions

Prolonged isolated thrombocytopenia (PT) includes primary poor platelet graft function (PPGF) and secondary failure of platelet recovery (SFPR) (1, 2). Patients with primary PPGF were defined as those who did not achieve initial platelet reconstitution, with persistent platelet counts below 20×10^9^/L or depended on PLT transfusions for more than 90 days after HSCT (1). SFPR was defined as a decline of platelet count to <50×10^9^/L for more than 7 consecutive days after initial platelet reconstitution (2). Patients with thrombocytopenia due to graft rejection or disease recurrence were not defined as PPGF, in accordance with the definition from a previous study (2). The date of platelet engraftment was defined as the first of 7 consecutive days with a platelet count of ≥20×10^9^/L, without transfusion support. Overall survival (OS) was defined from the time of transplant until death from any cause, or until the date of last follow-up. Relapse free survival (RFS) was defined from the time of transplant until disease relapse, or death from any cause, or until the date of last follow-up.

### Statistical Analysis

Categorical variables are shown as percentages and compared using the χ2 test. Continuous variables are presented as medians with interquartile ranges, and compared using Mann-Whitney U tests. Missing data were replaced using Random Forests in the ‘mice’ package of R, version 3.6.0 (http://www.r-project.org/). Cumulative incidence was visualized using Kaplan-Meier curves and compared using the log-rank test. Univariate and multivariate survival analyses for OS and RFS were undertaken by Cox proportional hazard models. The importance of individual variables was visualized using forest plots. Univariate analyses of risk factors were performed with univariate logistic regression. Risk factors with values of P <0.05 in the univariate analyses were chosen for further evaluation by multivariate logistic regression.

## Results

303 MDS patients who received allo-HSCT were included in our study. 184 (60.7%) were male and 119 (39.3%) were female. The median age of the cohort was 39 years (IQR 28-46). 107 (35.3%) patients received HLA matched sibling donors transplant, 69 (22.8%) received unrelated donors transplant, and 127 (41.9%) received haploidentical donors transplant. Before transplant, 76 (25.1%) achieved morphology-complete remission or complete remission. Most patients received MAC conditioning regimen (274 cases, 90.4%). After transplantation, 35 patients (11.6%) experienced primary PPGF, and 51 patients (16.8%) had SFPR. Acute GVHD (aGVHD) occurred in 150 (49.5%) patients. 100 patients (33.0%) had grade II–IV aGVHD and 35 patients (11.6%) extensive chronic GVHD (cGVHD). Cytomegalovirus (CMV) viremia was detected in 90 patients (29.7%) and Epstein-Barr virus (EBV) was identified in 43 patients (14.2%). Hemorrhagic cystitis (HC) occurred in 85 patients (28.1%) (**Table 1**).

**Table 1:** Patient characteristics.

Variables	No.	Good platelet graft function	Thrombocytopenia	P value
		No. (%)	No. (%)	
**Sex**				0.953
Male	184	132 (72)	52 (28)	
Female	119	85 (71)	34 (29)	
**Age**				0.512
< 40	153	107 (70)	46 (30)	
≥ 40	150	110 (73)	40 (27)	
**Blast**				0.990
< 5%	127	91 (72)	36 (28)	
≥ 5%	176	126 (72)	50 (28)	
**IPSS karyotype**				0.507
Good	171	118 (69)	53 (31)	
Intermediate	90	67 (74)	23 (26)	
Poor	42	32 (76)	10 (24)	
**WHO classification**				0.680
EB-1	81	55 (68)	26 (32)	
EB-2	98	72 (73)	26 (27)	
Others	124	90 (73)	34 (27)	
**IPSS**				0.666
Low	3	2 (67)	1 (33)	
Intermediate-1	165	118 (72)	47 (28)	
Intermediate-2	104	72 (69)	32 (31)	
High	31	25 (81)	6 (19)	
**IPSS-R**				0.959
Low	14	10 (71)	4 (29)	
Intermediate	83	60 (72)	23 (28)	
High	128	93 (73)	35 (27)	
Very high	78	54 (69)	24 (31)	
**Secondary MDS**				0.984
No	257	184 (72)	73 (28)	
Yes	46	33 (72)	13 (28)	
**Disease status before HSCT**				0.053
CR/mCR	76	61 (80)	15 (20)	
Others	227	156 (69)	71 (31)	
**Disease progression before HSCT**				0.196
No	240	176 (73)	64 (27)	
Yes	63	41 (65)	22 (35)	
**AML transformation before HSCT**				0.497
No	283	204 (72)	79 (28)	
Yes	20	13 (65)	7 (35)	
**Therapies before HSCT**				0.582
Supportive care	93	64 (69)	29 (31)	
DAC	96	73 (76)	23 (24)	
DAC + Chemotherapy	95	68 (72)	27 (28)	
Chemotherapy	19	12 (63)	7 (37)	
**Conditioning regimen**				0.334
RIC	29	23 (79)	6 (21)	
MAC	274	194 (71)	80 (29)	
**DAC in conditioning regimen**				0.120
No	198	136 (69)	62 (31)	
Yes	105	81 (77)	24 (23)	
**Using ATG in conditioning regimen**				0.774
No	88	62 (70)	26 (30)	
Yes	215	155 (72)	60 (28)	
**Donor type**				0.377
Sibling donor	107	78 (73)	29 (27)	
Unrelated donor	69	53 (77)	16 (23)	
Haploidentical donor	127	86 (68)	41 (32)	
**HLA typing**				0.396
10/10 or 6/6	166	124 (75)	42 (25)	
9/10	15	12 (80)	3 (20)	
6-8/10	31	21 (68)	10 (32)	
5/10	91	60 (66)	31 (34)	
**Source of stem cell**				0.756
BM	31	21 (68)	10 (32)	
PB	131	97 (74)	34 (26)	
BM+PB	140	98 (70)	42 (30)	
Cord	1	1 (100)	0 (0)	
**Gender of donor and receptors**				0.445
Male to male	112	84 (75)	28 (25)	
Male to female	75	51 (68)	24 (32)	
Female to male	72	48 (67)	24 (33)	
Female to female	44	34 (77)	10 (23)	
**ABO blood group of donor and receptors**				0.824
Matched	159	113 (71)	46 (29)	
Mismatched	144	104 (72)	40 (28)	
**GVHD prophylaxis**				0.851
CsA+MTX	94	68 (72)	26 (28)	
CsA+MMF+MTX	209	149 (71)	60 (29)	
**aGVHD**				**< 0.001**
No	153	128 (84)	25 (16)	
Yes	150	89 (59)	61 (41)	
**Grade of aGVHD**				**< 0.001**
None/I	203	164 (81)	39 (19)	
II-IV	100	53 (53)	47 (47)	
**cGVHD**				0.171
No	172	129 (75)	43 (25)	
Yes	131	88 (67)	43 (33)	
**Grade of cGVHD**				**0.016**
Others	268	198 (74)	70 (26)	
Extensive	35	19 (54)	16 (46)	
**HC**				**< 0.001**
No	218	172 (79)	46 (21)	
Yes	85	45 (53)	40 (47)	
**CMV infection**				**0.004**
No	213	163 (77)	50 (23)	
Yes	90	54 (60)	36 (40)	
**EBV infection**				0.080
No	260	191 (73)	69 (27)	
Yes	43	26 (60)	17 (40)	

IPSS, International prognostic scoring system; IPSS-R, revised IPSS; CR, complete remission; mCR, complete remission in morphology; DAC, decitabine; RIC, reduced intensive conditioning; MAC, myeloablative conditioning; ATG, anti-thymocyte globulin; HLA, human leukocyte antigen; GVHD, graft-versus-host disease; CsA, cyclosporine; MTX, methotrexate; MMF, mycophenolatemofetil; aGVHD, acute GVHD; cGVHD, chronic GVHD; HC, hemorrhagic cystitis; CMV, cytomegalovirus; EBV, Epstein-Barr virus. The P values in bold indicate statistical significance.

**Figure 1:**
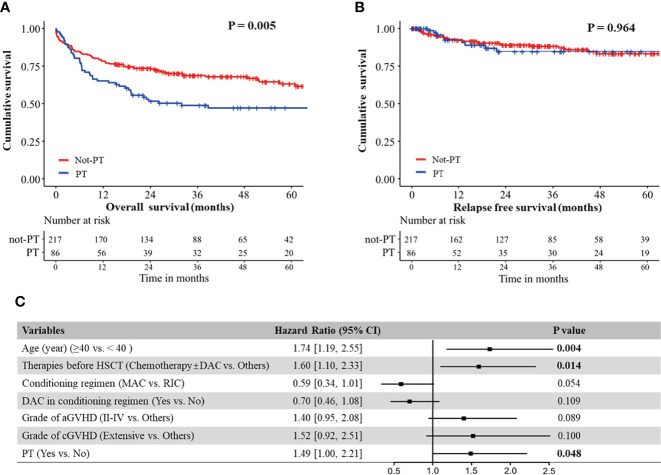
**(A)** Overall survival of MDS patients with and without PT; **(B)** Relapse free survival of MDS patients with and without PT. **(C)** Forest plot for hazard ratio of different risk factors on overall survival in MDS patients, as established using a Cox regression model.

Our analyses showed that OS of patients without PT was significantly better than that of patients with either primary PPGF (P = 0.033) or SFPR (P = 0.003), while no significant difference in OS was observed between the patients with primary PPGF and SFPR (P = 0.903) (**Supplementary Figure S1A**). However, for RFS, patients with primary PPGF or SFPR did not impact RFS in MDS patients after transplantation (**Supplementary Figure S1B**). Univariate analysis of risk factors affecting OS and RFS are listed in **Table 2**. Apart from older age, receiving chemotherapy before HSCT, receiving the RIC conditioning regimen, receiving a conditioning regimen without decitabine, grade II-IV aGVHD, and extensive cGVHD, PT was also a significant predictor of poor OS (**Figure 1**). The 2-year and 5-year OS in MDS patients with PT after HSCT were 49% and 47%, significantly worse than that of 68% and 60% in patients without PT (**Figure 1A**, P =0.005). However, for RFS, patients with PT did not have an increased risk of disease relapse, as shown in **Figure 1B** (P=0.964). After multivariate adjustment, PT was proved to be the independent risk factor associated with the worse OS (HR 1.49, 95% CI 1.00-2.21, P =0.048) (**Figure 1C**).

**Table 2:** Univariate analysis of OS and RFS in MDS patients who received HSCT.

Variables	Overall Survival			Relapse Free Survival		
	2-year (%)	5-year (%)	P value	2-year (%)	5-year (%)	P value
**Sex**			0.268			0.280
Male	59	53		86	81	
Female	69	61		91	82	
**Age**			**0.002**			**0.014**
< 40	70	69		90	86	
≥ 40	55	44		85	77	
**Blast**			0.274			0.202
< 5%	60	55		91	87	
≥ 5%	65	58		85	76	
**IPSS karyotype**			0.160			0.753
Good/int	64	57		88	81	
Poor	52	52		85	85	
**WHO classification**			0.087			0.310
Others	58	52		91	86	
EB-1/EB-2	66	59		86	77	
**IPSS**			0.452			0.186
Lower risk	66	58		91	86	
Higher risk	58	55		84	75	
**IPSS-R**			0.989			0.145
Lower risk	62	55		84	78	
Higher risk	63	57		89	82	
**Secondary MDS**			0.985			0.736
No	63	55		87	79	
Yes	60	60		88	88	
**Disease status before HSCT**			0.126			0.995
CR/mCR	67	67		86	73	
Others	61	54		88	83	
**Disease progression before HSCT**			0.388			0.292
No	64	58		89	81	
Yes	58	52		83	83	
**AML transformation before HSCT**			0.083			**0.001**
No	64	58		90	83	
Yes	43	35		61	61	
**Therapies before HSCT**			**0.005**			**0.018**
Others	69	64		90	83	
Chemotherapy ± DAC	52	44		86	81	
**Conditioning regimen**			**0.016**			0.910
RIC	45	45		87	87	
MAC	65	58		88	81	
**DAC in conditioning regimen**			**0.021**			0.221
No	59	51		86	79	
Yes	70	70		91	88	
**Using ATG in conditioning regimen**			0.307			0.585
No	58	52		89	86	
Yes	65	59		87	79	
**Donor type**			0.714			0.631
Sibling donor	64	57		89	85	
Others	62	57		87	79	
**Source of stem cell**			0.071			**0.029**
PB/PB+BM	65	58		89	82	
BM	48	44		71	71	
**Gender of donor and receptors**			0.778			**0.027**
Matched	61	56		82	79	
Mismatched	64	57		93	84	
**ABO blood group of donor and receptors**			0.653			0.289
Matched	64	61		85	77	
Mismatched	61	53		90	86	
**Gender of donors**			0.839			0.350
Male	59	59		88	88	
Female	65	55		87	77	
**Age of donors**			0.089			**< 0.001**
< 50	65	59		91	84	
≥ 50	46	40		66	60	
**GVHD prophylaxis**			0.556			0.517
CsA+MTX	66	57		90	85	
CsA+MMF+MTX	61	57		86	79	
**Grade of aGVHD**			**0.004**			0.050
None/I	69	61		85	80	
II-IV	51	46		93	85	
**Grade of cGVHD**			**0.049**			0.977
Others	66	58		88	81	
Extensive	42	42		83	83	
**HC**			0.180			0.557
No	66	58		88	79	
Yes	55	53		88	88	
**CMV infection**			0.291			0.291
No	62	53		87	77	
Yes	64	64		90	90	
**EBV infection**			0.922			**0.001**
No	63	57		91	85	
Yes	59	55		71	62	
**Thrombocytopenia**			**0.005**			0.964
No	68	60		88	79	
Yes	49	47		85	85	

IPSS, International prognostic scoring system; IPSS-R, revised IPSS; CR, complete remission; mCR, complete remission in morphology; DAC, decitabine; RIC, reduced intensive conditioning; MAC, myeloablative conditioning; ATG, anti-thymocyte globulin; HLA, human leukocyte antigen; GVHD, graft-versus-host disease; CsA, cyclosporine; MTX, methotrexate; MMF, mycophenolatemofetil; aGVHD, acute GVHD; cGVHD, chronic GVHD; HC, hemorrhagic cystitis; CMV, cytomegalovirus; EBV, Epstein-Barr virus. The P values in bold indicate statistical significance.

**Figure 2:**
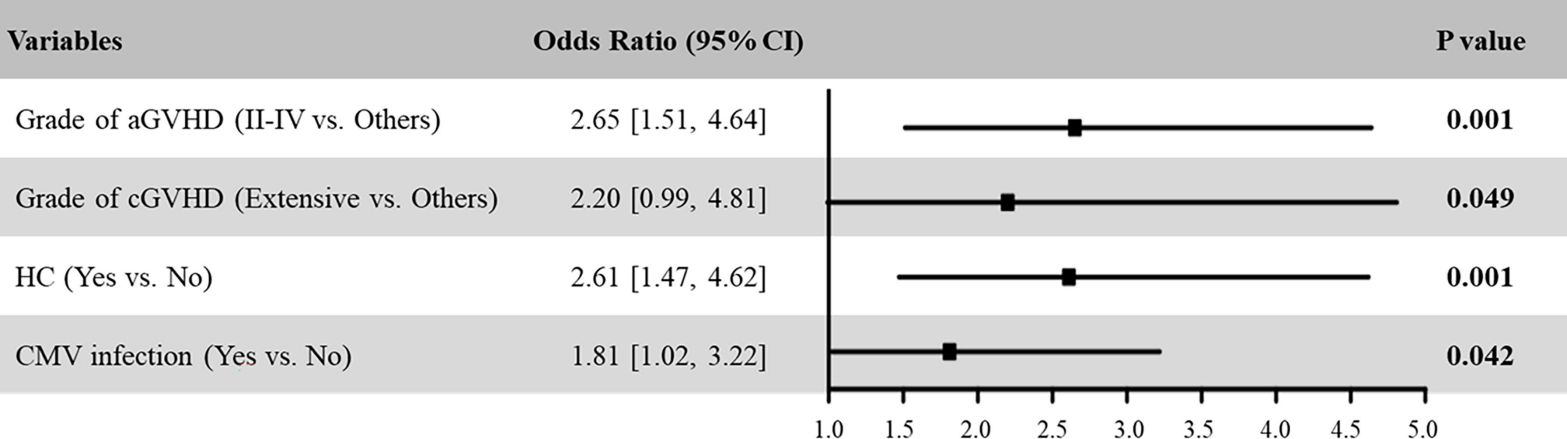
Multivariable logistic regression model assessing risk factors for PT in MDS patients after HSCT.

Being a significant complication after HSCT, patients with PT had inferior survival. We further analyzed risk factors associated with the occurrence of PT in MDS patients. Univariate analysis identified grade II-IV aGVHD, extensive cGVHD, HC, and CMV activation as risk factors for developing PT (**Table 3**). Including these variables in a multivariate logistic regression, the result showed that these four variables were the independent risk factors associated with the occurrence of PT in MDS after HSCT (**Figure 2A**). Of these, the OR of grade II-IV aGVHD was the greatest (P =0.001, OR: 2.65, 95% CI: 1.51-4.64).

**Table 3:** Univariate analysis of risk factors associated with thrombocytopenia in MDS after HSCT.

Variables	Non-adjusted Model		Adjusted Model	
	Odds Ratio [95% CI]	P value	Odds Ratio [95% CI]	P value
**Sex** (Female *vs*. Male)	1.02 [0.61, 1.69]	0.953	–	–
**Age** (year) (≥40 *vs*. < 40)	0.85 [0.51, 1.39]	0.512	–	–
**Blast** (≥5% *vs*. < 5%)	1.00 [0.61, 1.67]	0.990	1.02 [0.61, 1.70]	0.942
**WHO classification** (EB-1/2 *vs*. Others)	1.08 [0.65, 1.81]	0.757	1.10 [0.66, 1.85]	0.714
**IPSS** (Higher risk *vs*. Lower risk)	0.98 [0.59, 1.62]	0.935	0.99 [0.60, 1.64]	0.971
**IPSS-R** (Higher risk *vs*. Lower risk)	1.04 [0.61, 1.80]	0.885	1.04 [0.61, 1.80]	0.884
**Secondary MDS** (Yes *vs*. No)	0.99 [0.48, 1.95]	0.984	0.96 [0.46, 1.90]	0.909
**Disease status before HSCT** (Others *vs*. CR/mCR)	1.85 [1.01, 3.58]	0.056	1.88 [1.01, 3.65]	0.053
**Disease progression before HSCT** (Yes *vs*. No)	1.48 [0.81, 2.65]	0.198	1.49 [0.81, 2.67]	0.190
**AML transformation** **before HSCT** (Yes *vs*. No)	1.39 [0.51, 3.53]	0.499	1.42 [0.52, 3.62]	0.473
**Therapies before HSCT** (Chemotherapy ± DAC *vs*. Others)	1.12 [0.67, 1.87]	0.666	1.14 [0.68, 1.91]	0.615
**Conditioning regimen** (MAC *vs*. RIC)	1.58 [0.66, 4.41]	0.337	1.56 [0.65, 4.35]	0.354
**DAC in conditioning regimen** (Yes *vs*. No)	0.65 [0.37, 1.11]	0.122	0.65 [0.37, 1.11]	0.119
**Using ATG in conditioning regimen** (Yes *vs*. No)	0.92 [0.54, 1.61]	0.774	0.91 [0.53, 1.59]	0.733
**Transplant type** (MSDT *vs*. Others)	0.91 [0.53, 1.53]	0.715	0.92 [0.53, 1.56]	0.751
**Source of stem cell** (non-BM *vs*. BM)	0.81 [0.37, 1.88]	0.614	0.80 [0.37, 1.87]	0.594
**MNC dose** (×10^8^/kg) (≤ median *vs*. > median)	0.96 [0.58, 1.59]	0.884	0.96 [0.58, 1.58]	0.862
**CD34+ cell dose** (×10^6^/kg) (≤ median *vs*. > median)	1.29 [0.78, 2.13]	0.326	1.29 [0.78, 2.14]	0.320
**Gender of donor and receptors** (Mismatched *vs*. Matched)	1.53 [0.93, 2.55]	0.095	1.57 [0.94, 2.65]	0.086
**ABO blood group of donor** **and receptors** (Mismatched *vs*. Matched)	0.94 [0.57, 1.56]	0.824	0.95 [0.57, 1.57]	0.842
**Gender of donors** (Male *vs*. Female)	0.91 [0.55, 1.53]	0.721	0.91 [0.55, 1.52]	0.714
**Age of donors** (year) (≥ 50 *vs*. <50)	1.44 [0.66, 3.01]	0.345	1.45 [0.66, 3.04]	0.336
**GVHD prophylaxis** (CsA+MTX+MMF *vs*. CsA+MTX)	1.05 [0.62, 1.83]	0.851	1.04 [0.60, 1.82]	0.888
**aGVHD** (II-IV *vs*. Others)	3.73 [2.21, 6.34]	**< 0.001**	3.72 [2.20, 6.33]	**< 0.001**
**cGVHD** (Extensive *vs*. Others)	2.38 [1.15, 4.89]	**0.018**	2.36 [1.13, 4.89]	**0.020**
**HC** (Yes *vs*. No)	3.32 [1.95, 5.70]	**< 0.001**	3.31 [1.94, 5.68]	**< 0.001**
**CMV infection** (Yes *vs*. No)	2.17 [1.28, 3.69]	**0.004**	2.16 [1.27, 3.66]	**0.004**
**EBV infection** (Yes *vs*. No)	1.81 [0.91, 3.52]	0.083	1.79 [0.89, 3.53]	0.093

IPSS, International prognostic scoring system; IPSS-R, revised IPSS; CR, complete remission; mCR, complete remission in morphology; DAC, decitabine; RIC, reduced intensive conditioning; MAC, myeloablative conditioning; ATG, anti-thymocyte globulin; HLA, human leukocyte antigen; GVHD, graft-versus-host disease; CsA, cyclosporine; MTX, methotrexate; MMF, mycophenolatemofetil; aGVHD, acute GVHD; cGVHD, chronic GVHD; HC, hemorrhagic cystitis; CMV, cytomegalovirus; EBV, Epstein-Barr virus. The P values in bold indicate statistical significance.

Until last follow-up, 48 cases in PT group and 70 in non-PT group have died. Among these, 37(31%) were due to disease relapse. Of the 81 non-relapse deaths, infection and GVHD were either the main or contributing causes in patients with PT. And infection was the main cause of death in patients without PT. The causes of death in MDS patients after transplantation were shown in **Table 4**.

**Table 4:** Causes of death in MDS patients after HSCT.

	Thrombocytopenia (n=86)	Good platelet graft function (n=217)
**No. of death**	48	70
**Death from relapse**	10/48 (21%)	27/70 (39%)
**Deaths from causes other than relapse**		
**Infection**	17/48 (35%)	27/70 (39%)
**GVHD**	14/48 (29%)	3/70 (4%)
**Bleeding**	4/48 (8%)	4/70 (6%)
**Organ failure**	2/48 (4%)	6/70 (9%)
**Other**	1/48 (2%)	3/70 (4%)

## Discussion

PT is a serious complication post HSCT with a poor prognosis. Its reported incidence ranged from 3 to 50% following HSCT (1, 2, 4, 5, 7, 13). In our real-world study, we found an incidence of 28.4% for PT in MDS patients post HSCT. This variation may arise from the heterogeneity of criteria used to define PT (1–3, 12, 15, 16). Moreover, different patient selection in different centers may also contribute to differences in reported incidences. Most previous studies, including a report from our center, included several types of hematologic malignancy, including acute myeloid leukemia, acute lymphocytic leukemia, MDS, aplastic anemia, lymphoma and other disease type (1, 4, 5, 13, 16).

In this study, both grade II-IV aGVHD and extensive cGVHD were independent risk factors associated with the occurrence of PT in MDS patients post HSCT. Several previous studies reported correlations between aGVHD and platelet recovery after HSCT (2, 16, 17). Similar to the work of Kim et al., grade III-IV aGVHD was shown to be an independent risk factor for developing PT (16). The key mechanism involved in its development is thought to be GVHD-related autoimmune destruction (8). Platelet autoantibodies have been observed in patients after both autologous HSCT and allo-HSCT (7, 8, 18). According to the report from Anasetti C et al., platelet autoantibodies were only seen patients with GVHD, whereas in patients without GVHD, autoantibodies were not observed (8). Yamazaki R et al. indicated that in addition to antiplatelet antibody, reticuloendothelial system, which was damaged by GVHD, was also implicated in the development of PT (7).

CMV infection is another common complication, causing morbidity and mortality after HSCT. Consistent with previous studies, CMV infection has been suggested to be correlated with PT after HSCT (2, 4, 19). The role of CMV infection in the pathophysiology of PT is not fully understood. Several *in vitro* studies have shown that early hematopoietic progenitors are more susceptible to CMV infection, resulting in the inhibition of their proliferative function (20, 21). Apart from the direct cytotoxicity of CMV in hematopoietic progenitor cells, CMV-related impairment of stromal function, abnormal gene expression, and the indirect immune destruction of CMV infected hematopoietic cells have all been suggested as pathological mechanisms underlying PT development (22–25). In addition, Crapnell et al. have shown that differentiated megakaryocytes and their precursors are targets of CMV infection *in vitro*, contributing to thrombocytopenia (26).

Our study supported HC as an independent risk factor predictive of PT development of PT post HSCT. The correlation of platelet recovery and HC has been evaluated in several studies, and the results are controversial (27–29). Lunde et al. observed that HC resolution is associated with raised platelet counts (27). However, other studies suggest platelet counts are maintained > 50 × 10^9^/L in patients with active HC (30–32). Because acute or chronic GVHD and HC may exist or that immunosuppressive therapies used to treat GVHD increase can the probability of opportunistic infections which subsequently cause HC (33, 34). Other studies have suggested an association between CMV reactivation and HC (35, 36), as DNA viruses may induce BK virus Replication (37, 38). The involvement in PT of both GVHD and CMV have been suggested, with different mechanisms as discussed above. A complex relationship exists amongst HC, GVHD, and CMV infection, and it is reasonable that HC was identified as a risk factor for PT in MDS patients post HSCT.

In conclusion, our results indicate that PT predicts poor OS in MDS patients after HSCT. The identification of risk factors for PT may help clinicians to more accurately assess the prognosis and design new treatment strategies.

## Data Availability Statement

The original data presented in this study is available on request from the corresponding author hanyue@suda.edu.cn.

## Ethics Statement

This study was reviewed and approved by Ethics Committee of the First affiliated Hospital of Soochow University. Participants provided their written informed consent to participate in this study.

## Author Contributions

HW, JQ, XL, TC: contribution of patients, acquisition of data, analysis and interpretation of data. YH, DW and HW: Design of study, acquisition of funding contribution of patients, interpretation of data, supervision of the study, and revision of the manuscript. HQ, CF, XT and CR: contribution of patients and revision of the manuscript. HW, JQ and XL wrote the paper. All authors contributed to the article and approved the submitted version.

## Funding

This work was supported by National Natural Science Foundation of China (81100342, 81873432 and 82070143), grants from the Jiangsu Province of China (BE2021645), and the Priority Academic Program Development of Jiangsu Higher Education Institutions (PAPD).

## Conflict of Interest

The authors declare that the research was conducted in the absence of any commercial or financial relationships that could be construed as a potential conflict of interest.

## Publisher’s Note

All claims expressed in this article are solely those of the authors and do not necessarily represent those of their affiliated organizations, or those of the publisher, the editors and the reviewers. Any product that may be evaluated in this article, or claim that may be made by its manufacturer, is not guaranteed or endorsed by the publisher.
