# Supplementary material for: Prognostic Value of Thrombocytopenia in Myelodysplastic Syndromes After Hematopoietic Stem Cell Transplantation
Source: Front Oncol. 2022 Jul 11;12:940320. doi: 10.3389/fonc.2022.940320 (PMC9309887; doi:10.3389/fonc.2022.940320)
Supplement: Supplementary file 1 [file DataSheet_1.docx]

**Supplementary Figure S1**

**Supplementary Figure S1.** (**A)** Overall survival of MDS patients according to platelet graft function; (**B)** Relapse free survival of MDS patients according to platelet graft function. PT: prolonged isolated thrombocytopenia; primary PPGF: primary poor platelet graft function; SFPR: secondary failure of platelet recovery.
